# History of the Life of D. Hayes Agnew, M. D., L. L. D.

**Published:** 1893-05

**Authors:** 


HISTORY OF THE LIFE OF D. HAYES AGMEW, M. D., LL.D. By J. Howe Adams, M. D. With fourteen full-page portraits and other illustrations. In one large Royal Octavo volume, 376 pages, Extra Cloth, bevelededges, $2.50 net ; Half-Morocco, gilt top, $3.50 net. *Sold only by Subscription.* Philadelphia : The F. A. Davis Co., Publishers, 1914 and 1916 Cherry Street.

Few books are more entertaining than a well written biography. The life of Dr. Agnew was full of incidents calculated to afford the biographer ample material In the volume before us, this has been well used and a most entertaining book has been the result.

